# The ‘other’ colonic polyposis syndromes—evolving insights and guidance for endoscopists

**DOI:** 10.1007/s00384-026-05186-8

**Published:** 2026-06-25

**Authors:** Amanda Liesegang, Andy Wu, Alexander Heriot, Glen Guerra

**Affiliations:** 1 Sir Peter MacCallum Department of Oncology, University of Melbourne, 305 Grattan Street, Melbourne, VIC 3000 Australia; 2 Division of Cancer Surgery, Peter MacCallum Cancer Centre, 305 Grattan Street, Melbourne, VIC 3000 Australia; 3 Northern Health Service, Melbourne, VIC Australia

**Keywords:** Polyposis syndromes, Colonic polyps, Endoscopic surveillance, Mismatch repair deficiency, Colorectal neoplasia

## Abstract

**Purpose:**

Polyposis syndromes contribute to a significant proportion of colorectal cancer (CRC) diagnoses. While Lynch syndrome (LS) and familial adenomatous polyposis (FAP) are well characterised, a growing number of rarer polyposis syndromes are increasingly recognised in clinical practice. These conditions often present with overlapping phenotypes and variable penetrance, posing diagnostic and management challenges for the practising clinician. The evidence base remains limited, with current guidance largely derived from small cohort studies and expert opinion. This narrative review summarises the current literature on the genetic basis, clinical manifestations, and endoscopic features of the rarer colorectal polyposis syndromes, excluding LS and FAP.

**Methods:**

A comprehensive literature search was conducted using PubMed and Ovid databases. Selected articles were evaluated for relevance and quality, and key findings were synthesised narratively to provide an overview of current knowledge and emerging trends relating to the rarer polyposis syndromes.

**Results:**

This review consolidates international recommendations for surveillance and management, with a focus on practical guidance for the endoscopist. Advances in next-generation sequencing and multigene panel testing have reshaped our understanding of polyposis genetics, leading to the identification of several novel autosomal dominant and recessive syndromes. Despite these discoveries, surveillance protocols remain heterogeneous, and gaps persist in defining cancer risk, optimal timing of intervention, and the role of chemoprevention. Greater awareness of these syndromes among clinicians is essential for timely diagnosis and personalised management.

**Conclusion:**

Collaborative registries, prospective data, and consensus-driven guidelines are urgently required to standardise care and improve outcomes for patients with rare polyposis syndromes.

**Supplementary Information:**

The online version contains supplementary material available at https://doi.org/10.1007/s00384-026-05186-8.

## Introduction

Colorectal cancer (CRC) is the third most common malignancy and the second leading cause of cancer-related mortality worldwide [1]. In recent years, rising rates of early-onset CRC have emerged as a significant public health concern, with increasing incidence reported in 27 countries, particularly across high-income Western regions [2]. Familial colorectal cancer syndromes contribute to 5–10% of all CRC diagnoses, with familial adenomatous polyposis (FAP) and Lynch syndrome (LS) being the most prevalent and well-characterised entities [3]. However, several less common hereditary syndromes have also been identified, and substantial progress has recently been made in elucidating their molecular basis, underlying pathophysiology, and clinical phenotypes.

Despite these advancements, there remains a paucity of long-term prospective data assessing the efficacy of different management and surveillance strategies for these conditions [4]. Consequently, consensus statements from international expert panels continue to form the foundation of current recommendations, which are frequently updated in light of new genetic and clinical insights. This review provides a comprehensive synthesis of current evidence regarding the pathogenesis, genetic basis, and clinical features of the ‘other’ hereditary syndromes (excluding FAP and LS). It also summarises current management and surveillance recommendations, serving as a practical guide for the endoscopist. Additionally, the review highlights key gaps in knowledge and proposes directions for future research. For clarity, these syndromes are categorised as follows: (1) serrated, (2) hamartomatous, (3) adenomatous—autosomal dominant, (4) adenomatous—autosomal recessive, (5) mismatch repair deficiency-related, and (6) mixed polyposis syndromes.

## Methodology

This narrative review was conducted to identify and summarise the existing literature on the rarer colonic polyposis syndromes. A formal systematic review of this topic was not a suitable approach, due to the paucity of long-term data from high-quality primary studies currently presented in the field. The narrative review approach allowed for broad examination of the rarer syndromes with emerging evidence in the literature. A literature search was performed in December 2024 using both electronic databases PubMed and Ovid (MEDLINE), searching for articles published between 2010 and 2024. Keywords search terms included combinations of keywords: cancer, polyposis, polyp, syndrome, hereditary, colorectal, genetics, surveillance, colonoscopy, management, and guidelines. Boolean operators (OR, AND) were used to refine the search. Articles were included for review if they were peer-reviewed articles/reports which discussed any colonic polyposis syndrome, with particular emphasis on the incidence, genetic basis, surveillance recommendations, and management guidelines for these conditions. Only studies published in English were included. Articles excluded from the study consisted of case reports, articles focused on FAP/LS, and articles providing generalised surveillance guidelines not specific for any particular condition. Reference lists of included articles were manually screened to identify additional relevant studies, and key authors in the field were reviewed to ensure comprehensive coverage. The literature search was conducted by authors AL, AW, and GG. Titles and abstracts were screened for relevance. Full texts of potentially relevant articles were then reviewed. Selection was based on relevance to the review objectives and contribution to identified syndromes. Studies were grouped by identified syndromes, and these syndromes were subsequently grouped by genotypic/phenotypic features to provide a coherent narrative of the topic. The groups included: serrated, hamartomatous, adenomatous—autosomal dominant, adenomatous—autosomal recessive, mismatch repair deficiency-related, and mixed polyposis syndromes.

## Serrated polyposis syndromes

### Serrated polyposis syndrome (SPS)

#### Diagnosis

SPS (formerly hyperplastic polyposis syndrome) predisposes patients to multiple large, serrated polyps in the colon, increasing the risk of colonic malignancy. Although previously considered rare, prevalence is 1:111 in screening populations, making it now the most common polyposis syndrome [5]. The WHO 5th edition diagnostic criteria for SPS, recently updated in 2019, require one of the following [6]:

i.  ≥ 5 serrated polyps proximal to the rectum, all ≥ 5 mm, with ≥ 2 being ≥ 10 mm
ii.  > 20 serrated polyps of any size, ≥ 5 proximal to the rectum.

The polyp count includes any type of serrated polyps and is cumulative across lifetime colonoscopies.

#### Genetics and cancer risk

CRC in SPS is genetically and clinically heterogeneous. Various pathways, such as somatic *BRAF* mutations, microsatellite instability (MSI), and CpG island methylator phenotype, can all contribute to serrated polyp pathogenesis [7]. While a small proportion of cases carry a germline mutation in the *RNF43* gene, it remains uncommon, so genetic testing is not routinely recommended. Diagnosis relies on fulfillment of the WHO criteria [8]. Historical CRC risk estimates in SPS have varied widely, between 10 and 70% in the literature [3, 8–11]. A recent meta-analysis including 36 studies concluded the overall rate of CRC in SPS patients may be more accurately around 19.9% (95% confidence interval, 15.3–24.5%) [12].

#### Endoscopic/histological features

There are three main types of sessile serrated lesions: hyperplastic polyps (HPs), sessile serrated lesions (SSLs), and traditional serrated adenomas (TSAs). HPs are the most common, typically < 5 mm with a nodular pale appearance and located in the rectosigmoid colon [13]. In isolation, they do not exert an increased risk of CRC [14]. SSLs, found mainly in the proximal colon, are often flat and hard to detect. They are frequently under-recognised due to their flat profile, indistinct borders, and propensity to be obscured between mucosal folds or under a mucus cap. TSAs are the largest and rarest type of serrated polyps, often multilobulated, slightly erythematous, and more prevalent in the rectosigmoid colon. All SSLs have serrated crypt architecture, but differ in structure, dysplastic potential, and sites of predilection.

#### Surveillance/management

Management of SPS should include colonoscopy every 1–3 years to remove all ‘relevant’ polyps: ≥ 5 mm polyps, and all polyps of any size with macroscopic dysplastic features. Once cleared of ‘relevant’ polyps, surveillance should be tailored to the individual based on findings at their last colonoscopy. If ≥ 1 advanced polyp is found (including any polyp ≥ 10 mm, SSLs with dysplasia, TSAs, or non-serrated adenomatous polyps with high-grade dysplasia or ≥ 25% villous histology), or ≥ 5 non-advanced ‘relevant’ polyps are found, yearly surveillance is recommended. Two to 3 yearly surveillance is advised otherwise [9–11, 15–21]. If CRC is diagnosed, extended resection can be performed for risk reduction [3, 8, 9]. First-degree relatives (FDRs) should begin screening at age 40 or 10 years before age of diagnosis of the index case of SPS in the family (whichever is youngest). They should undergo repeat screening every 5 years unless polyps are detected [8, 10, 18, 22, 23]. Limited data exists regarding the risk of extracolonic cancers in patients with serrated polyposis syndrome (SPS). While some studies have reported a risk of 24–28%, these findings have not been consistently replicated nor compared to the general population, and consequently, routine screening for other cancer types is not currently recommended [24].

## Hamartomatous polyposis syndromes

### Peutz-Jeghers syndrome (PJS)

#### Diagnosis

PJS is an autosomal dominant syndrome characterised by the development of hamartomatous polyps of the gastrointestinal tract (GIT) and perioral mucocutaneous pigmentation. It is associated with an increased risk of both gastrointestinal and non-gastrointestinal malignancies. Incidence is estimated to be 1:8000–1:200,000 [5]. The mucocutaneous pigments appear in early childhood and are seen in 95% of patients [25]. Hamartomas are generally found in the small bowel, colon, and stomach. They begin to develop during childhood and can cause obstruction, infarction, anaemia, or intussusception, generally in the second to third decades of life [26].

PJS is diagnosed when any one of the following criteria is present [4]:

-  ≥ 2 Peutz-Jeghers polyps in the GIT
- Mucocutaneous hyperpigmentation of the mouth, lips, nose, eyes, genitalia, or fingers in a person with family history of PJS
-  ≥ 1 Peutz-Jeghers polyp in an individual with family history of PJS in an FDR
-  ≥ 1 Peutz-Jeghers polyp in patients with characteristic mucocutaneous pigmentation

#### Genetics and cancer risk

PJS follows an autosomal dominant inheritance pattern, with 80–94% of patients having a germline mutation in the *STK11* gene [26, 27]. Those without a detectable *STK11* pathological variant may have a less severe presentation of the disease. This tumour suppressor gene regulates multiple pathways, including the mTOR pathway which is also dysregulated in other hamartomatous syndromes. Genetic testing for *STK11* mutation is advised for patients who meet the above criteria and for the children of affected patients if the diagnosis is not already clinically apparent by 8 years of age [3, 4, 26]. PJS patients face a lifetime malignancy risk of 85% [10, 26], with significantly increased risks of malignancy in the colorectum (39%), stomach (29%), small bowel (13%), pancreas (11–36%), breast (32–54%), and testicles (9%) [28].

#### Endoscopic/histological features

PJ hamartomas are not endoscopically distinct and often vary in size and distribution. They can be lobulated, sessile, or pedunculated, with patients often found to have a combination of these on endoscopic examination. Histologically, they can be identified via features like cystic gland dilatation and smooth muscle arborization, with often normal overlying epithelium [3, 4, 26, 27].

#### Surveillance/management

Given the high risk of multi-organ malignancies, multidisciplinary surveillance via a familial cancer clinic is essential for management of PJS. PJS polyps rarely undergo malignant transformation prior to adulthood; therefore, childhood management is focused on preventing complications, and in adulthood, it shifts towards surveillance and risk reduction [4, 26]. Baseline endoscopy, colonoscopy, and video capsule endoscopy (VCE) are recommended at age 8 and again at 18 if no polyps are identified [3, 4, 10, 15, 25, 27, 29, 30]. Surveillance should be performed every 1–3 years thereafter depending on findings from prior investigations. Extracolonic surveillance recommendations are shown in Table 1. Polypectomy is advised for all polyps > 5 mm in the stomach and colon and > 10 mm in the small intestine. Bowel resection with consideration of extension for risk reduction is recommended for malignant polyps [3, 4, 26, 31]. Emergency surgery may also be necessary in the setting of acute intussusception of the small intestine. Concurrent on-table enteroscopy and polyp clearance are recommended if appropriate during these operations [4]. The potential role of COX2-inhibitors as chemoprophylaxis has been explored in mouse models; however, there remains insufficient evidence for routine application in humans yet [10, 27].

**Table 1:** Cancer risk, average age of diagnosis, and surveillance recommendations for polyposis syndromes

Syndrome + cancer site	Cancer risk, *%*	Average age of Dx, *y*	Surveillance starting age, *y*	Surveillance recommendations	References
*1.1 Serrated polyposis syndrome*					
Colon	~ 20%	40–60	From diagnosis 40 (FDR)^a^	3–6 monthly colonoscopy initially 1–3 yearly colonoscopy thereafter 5 yearly colonoscopy	[3, 8–12, 15–19, 21, 29] [8, 10, 18, 22]
*2.1 Peutz-Jeghers syndrome*	Overall—85%^b^				
Colon	39%	42–46	8, 18^c^	1–3 yearly colonoscopy	[3, 4, 10, 15, 27, 30]
Stomach	29%	30–40	8, 18^c^ 8	1–3 yearly OGD 1–3 yearly VCE, MRE	[3, 4, 10, 15, 25, 27, 30]
Small bowel	13%	37–42	8, 18^c^ 8	1–3 yearly OGD 1–3 yearly VCE, MRE	[3, 4, 10, 15, 25, 27, 30]
Pancreas	11–36%	41–52	25–35	1–3 yearly EUS, MRCP, MRI	[3, 4, 19, 27, 29, 55]
Breast	32–54%	37–59	18 25	Annual self-examination Annual mammogram, MRI	[3, 4, 19, 22, 27, 29, 30, 55]
Ovarian	21%	28	18–25	Annual pelvic examination, TVU	[3, 4, 19, 27, 29, 30, 55]
Endometrial	9%	43	18–25 18	Annual pelvic examination, TVU 2 yearly hysteroscopy	[3, 4, 19, 27, 29, 30, 55]
Cervix	10–23%	34–40	18–25	1–2 yearly pelvic examination, pap smear	[3, 4, 19, 27, 29, 30, 55]
Testicular	9%	6–9	Birth–teenage	Annual testicular exam, US^d^	[3, 4, 19, 27, 29, 55]
Lung	7–17%	47	-	Symptom education + smoking cessation	[3, 4, 19, 27, 29, 55]
*2.2 Juvenile polyposis syndrome*	Overall: 70–90%^b^				
Colon	68%	44 (34–47)	12–15	2–3 yearly colonoscopy	[3, 4, 10, 15, 22, 23, 25, 27, 31, 60]
Upper GI-stomach	10–25%	41–58	12–15	2–3 yearly OGD	[3, 4, 10, 15, 22, 23, 25, 27, 31, 60]
Small bowel (duodenum)	10–25%	-	12–15	2–3 yearly CT/MRE, OGD	[15, 33]
*2.3 Cowden’s syndrome*					
Colon	9–16%	44–58	35–40	2–3 yearly colonoscopy if positive findings Otherwise as per general population thereafter	[3, 4, 22, 27, 29, 44, 80]
Upper GI	5–20%	44–48	15	2–3 yearly OGD	[3, 27, 29, 80]
Breast (most common)	25–85%	38–46	30–35 30–40 25 25	Annual MRI Annual mammogram Monthly breast self-exam 6–12 monthly clinical breast exam	[3, 4, 29, 44, 60, 80]
Thyroid	3–38%	31–38	12	Annual thyroid exam and US	[3, 4, 29, 44, 60, 80]
Endometrial	5–25%	25	30–35	1–2 yearly endometrial sampling, TVU	[3, 4, 29, 60, 80]
Kidney	15–34%	40	40	1–2 yearly urinalysis and cytology +/− Renal US	[3, 4, 29, 80]
Melanoma	6%	3^e^	At dx, by 18	Annual skin examination	[3, 4, 29, 80]
*3.1 Polymerase proofreading-associated polyposis*					
Colon				1–2 yearly colonoscopy	[46, 47, 49]
POLE gene	21–90%		14–25		
POLD1 gene	44–90%				
Upper GI	-	-	20–25	1–3 yearly OGD	[47, 49]
Endometrial	-	-	40	Endometrial cancer screening	[36, 38]
Ovarian	-	-	-	Discuss risk reducing hysterectomy	[47]
Breast	20–30%	-	-	Refer to high-risk breast screen	[36, 38]
*3.2 Oligodontia-colorectal cancer syndrome*					
Colon	-	-	25–30	2–3 yearly colonoscopy	[51–53]
Upper GI	-	-	25–30	1–3 yearly OGD	[51–53]
*4.1 MUTYH-associated polyposis*					
Colon	43–63%	45–50	18–20 FDR—45	1–2 yearly colonoscopy 5 yearly colonoscopy	[3, 10, 15, 19, 22, 27, 29, 50, 55, 56, 60]
Upper GI	1–6%	50 +	20–35	1–3 yearly OGD	[3, 10, 15, 19, 22, 27, 29, 50, 55, 56, 60]
*4.2 NTHL1 tumour syndrome*					
Colon	Likely high	31–73	18–20	2 yearly colonoscopy	[50, 62–64]
Upper GI	-	-	25	5 yearly OGD	[50, 62–64]
Breast	-	36–63	30	1–2 yearly mammogram/MRI	[50, 62–64]
Endometrial	-	47–74	40	2 yearly endometrial sampling, TVU	[50, 62–64]
*4.3 MBD4-associated neoplasia syndrome*					
Colon	Likely high	20–30 s	18–20	2 yearly colonoscopy	[47, 50, 65]
Haematological	-	-	From Dx	Annual full blood count	[47, 50, 65]
Ophthalmologic	-	-	From Dx	Annual ophthalmologic assessment	[47, 50, 65]
*4.4 MSH3-associated polyposis*					
Colon	-	20–30	18–20	1–2 yearly colonoscopy	[66–69]
*4.5 MLH3-associated polyposis*					
Colon	-	40–50	-	Colonoscopy	Insufficient data
*5.1 Constitutional mismatch repair deficiency *^*f*^					
Colon	-	-	6–8	Annual colonoscopy	[72, 75–77]
Upper GI	-	-	8–10	Annual gastroscopy	[72, 75–77]

*Dx* diagnosis, *ECG* electrocardiogram, *EUS* endoscopic ultrasound, *FDR* first degree relative, *HHT* hereditary haemorrhagic telangiectasia, *MRCP* magnetic resonance cholangiopancreatography, *MRE* magnetic resonance enterography, *MRI* magnetic resonance imaging, *OGD* oesophagogastroduodenoscopy, *TTE* transthoracic echocardiogram, *TVU* transvaginal ultrasound, *US* ultrasound, *VCE* video capsule endoscopy

^a^Family screening should start at 40 yo or 10 yrs prior to index case, whichever is youngest

^b^Risk of ALL cancers

^c^Baseline colonoscopy at 8 yo, repeat at 18 yo if negative and every 1–3yrs thereafter

^d^US performed if feminisation or other abnormality detected

^e^Age of the youngest patient found with melanoma

^f^Weak evidence, protocols based on current estimates of malignancy risks

### Juvenile polyposis syndrome (JPS)

#### Diagnosis

JPS is characterised by the formation of juvenile hamartomatous polyps within the GIT. It presents in either early childhood (‘juvenile polyposis of infancy’) or early adulthood (‘juvenile polyposis coli’ and ‘generalised juvenile polyposis’). ‘Juvenile polyposis of infancy’ is rare and severe and can be associated with malrotation, cleft palate, and polydactyly. It has a high mortality rate in comparison to the other two latter variants, where patients typically present in the second decade of life with GI bleeding and anaemia [32]. Extra-intestinal manifestations of JPS include hereditary haemorrhagic telangiectasia (HHT), mitral valve prolapse, arterial aneurysms, and skeletal and cranial abnormalities [33]. Incidence is estimated at 1:100,000 to 1:160,000 [34].

The WHO criteria for JPS diagnosis require one of the following [4, 33]:

-  ≥ 5 juvenile polyps in the colon/rectum
-  ≥ 2 juvenile extracolonic polyps
- Any number of juvenile polyps AND ≥ 1 FDR with JPS

Patients who meet these criteria should undergo genetic evaluation, since the underlying variant can impact phenotypic expression and assist in differentiating this condition from other hamartomatous polyposis syndromes [10, 31, 33].

#### Genetics and cancer risk

JPS is autosomal dominant, with germline mutations in *SMAD4* or *BMPR1A* genes accounting for 40–60% of cases [35, 36]. *SMAD4* mutations are associated with more aggressive disease, characterised by earlier cancer development, aggressive gastric polyposis, and hereditary haemorrhagic telangiectasia (HHT) [25, 33]. In contrast, *BMPR1A*-mutated patients have less gastric involvement and overall less aggressive polyp formation. JPS patients have a CRC risk of 68% by age 60 and a 10–25% lifetime risk for gastric and duodenal malignancy [37].

#### Endoscopic/histological features

Juvenile polyps are hamartomatous and commonly found in the colon, stomach, and small bowel. On endoscopy, they can be sessile or pedunculated with a smooth red surface. Histologically, they show nondysplastic epithelium and cystic glands with chronic inflammatory infiltrate [4, 33, 35]. Fifty percent of JPs have adenomatous change [34].

#### Surveillance/management

The main goal of JPS management should be to decrease the bleeding risk and the recurrence of gastric and colorectal cancers. Surveillance should begin at age 12–15, with annual colonoscopy and gastroscopy until all polyps are cleared. Subsequent surveillance continues every 2–3 years depending on disease severity [4, 15, 25, 33–36]. VCE is not routinely indicated outside of symptomatic or anaemic patients [35]. As is the case with PJS, chemoprophylaxis remains an area of continued research for JPS cancer risk management; however, there is yet to be any proven benefits thus far [10, 27]. For *SMAD4* mutation carriers, additional screening for pulmonary and cerebral AVMs, valvular dysfunction, and aortic disease is recommended [10, 33–35]. Endoscopic polypectomy should be performed to remove colorectal polyps > 10 mm, while colectomy can be considered for refractory bleeding, malignancy, or if polyp burden is not manageable endoscopically. Fifty percent of those who undergo a colectomy and ileorectal anastomosis will require completion proctectomy in their lifetime [3].

### Cowden’s syndrome (CS)

#### Diagnosis

CS is a phenotypic variation of PTEN-hamartoma tumour syndrome (PHTS), which describes a group of phosphatase and tensin homolog gene (*PTEN*) gene-related disorders. It is a rare autosomal dominant condition characterised by hamartomatous polyps and diffuse oesophageal glycogenic acanthosis [3, 22, 27, 38]. It also presents with extra-intestinal manifestations such as mucocutaneous lesions, macrocephaly, and Lhermitte-Duclos disease—a rare slow-growing tumour of the cerebellum [31]. The estimated prevalence is 1:200,000 to 250,000 [39]. The clinical criteria for CS diagnosis are complex and have been continually revised, with the International Cowden Consortium and Pilarski team (adopted by the National Comprehensive Cancer Network- NCCN) proposing diagnostic criteria outlined in Table 2 [38, 40, 41].

**Table 2:** Indications and criteria for genetic screening of Cowden’s syndrome [38]

Indications for genetic evaluation	Major criteria	Minor criteria
Individual from a family with a known *PTEN* mutation	Breast cancer	Autism spectrum disorder
Individual meeting clincal diagnostic criteria for CS	Endometrial cancer	Colon cancer
Indivual with a personal history of any of:	Follicular thyroid cancer	Oesophageal glycogenic acanthosis (≥ 3)
Bannayan-Riley-Ruvalcaba Syndrome (BRRS)	Multiple GI hamartomas or ganglioneuromas	Lipomas
Adult Lhermitte-Duclos disease	Macrocephaly	Mental retardation (IQ < 75)
Autism spectrum disorder and macrocephay	Macular pigmentation of glans penis	Papillary or follicular variant of papillary thyroid cancer
Two or more biopsy proven trichilemmomas	Mucocutaneous lesions alone:	Thyroid structural lesions (adenoma, nodules, goitre)
Two or more major criteria (one must be macrocephaly)	One biopsy proven trichilemmoma	Renal cell carcinoma
Three major criteria, without macrocephaly	Multiple palmoplantar keratoses	Single gastrointestinal hamartoma or ganglioneuroma
One major and ≥ three minor criteria	Multifocal or extensive oral papillomatosis	Testicular lipomatosis
≥ Four minor criteria	Multiple cutaneous facial papules (Verrucous)	Vascular anomalies
At risk individual with one major or two minor criteria and a relative with a clinical diagnosis of CS/BRRS (Not tested)		

Diagnosis requires the following: (1) three or more major criteria; (2) two major and three minor criteria; and (3) PHTS diagnosis in a family member and (A) any two major criteria, (B) one major and two minor criteria, or (c) three minor criteria

#### Genetics and cancer risk

Germline mutation in the *PTEN* tumour suppressor gene is responsible for CS, though only 30% of those meeting clinical criteria harbour a detectable *PTEN* mutation, and 30% are due to de novo mutations (Table 3) [3]. In those with CS who lack a detectable *PTEN* mutation, germline mutation of the *KLLN*, *SDH B/D*, or *AKT1* genes or somatic mutation in the *PIK3CA* gene can be the cause [42]. CS increases the risk of several cancers, including breast, thyroid, endometrial, skin, colon, and kidney cancers, with a lifetime risk of breast cancer ranging from 25 to 85% [29, 31]. The lifetime malignancy for other organs is summarised in Table 1.

**Table 3:** Polyposis syndrome genetics and phenotype

Polyposis type	Syndrome	Gene(s) involved	Inheritance	Main polyp type(s)	Polyp numbers
Serrated	Serrated Polyposis syndrome	RNF43*	Unknown	Serrated Hyperplastic	2–200
Hamartomatous	Peutz-Jeghers syndrome	*STK11*	Autosomal dominant	Hamartomatous	1–10
	Juvenile polyposis	*SMAD4* *BMPR1A*	Autosomal dominant	Hamartomatous	< 10–100
	Cowden’s syndrome	*PTEN*	Autosomal dominant	Hamartomatous	0–100
Adenomatous	Polymerase proofreading-associated polyposis	*POLE* *POLD1*	Autosomal dominant	Adenomatous	10–100
	Oligodontia-colorectal cancer syndrome	*AXIN2*	Autosomal dominant	Adenomatous	10–100
	*MUTYH*-associated polyposis	*MUTYH*	Autosomal recessive	Adenomatous Serrated	10–100
	*NTHL1* tumour syndrome	*NTHL1*	Autosomal recessive	Adenomatous	2–200
	*MBD4*-associated neoplasia syndrome	*MBD4*	Autosomal recessive	Adenomatous	10–100
	*MSH3*-associated polyposis	*MSH3*	Autosomal recessive	Adenomatous	10–100
	*MLH3*-associated polyposis	*MLH3*	Autosomal recessive	Adenomatous	50–200
Mismatch-repair deficiency related	Constitutional mismatch repair deficiency	*MLH1, MSH2, MSH6, PMS2*	Autosomal recessive	Mixed	< 100
Mixed	Hereditary mixed polyposis syndrome	*GREM1*	Autosomal dominant	Hyperplastic, atypical juvenile, adenomatous	10–100

^*^This mutation has only been found in a small minority of patients and is unlikely to be a major driver of SPS pathogenesis

#### Endoscopic/histological features

Colonic polyps are quite commonly found in CS patients. These are commonly hamartomatous in nature but may also include adenomas, ganglioneuromas, leiomyomas, juvenile polyps, inflammatory polyps, or lipomas [3]. A typical finding during gastroscopy is diffuse glycogenic acanthosis of the oesophagus, which is a minor diagnostic criterion [43].

#### Surveillance/management

Patients with suspected CS should undergo genetic testing for *PTEN* mutation, and this should be extended to family once confirmed. A multi-disciplinary approach to cancer surveillance is required to address the high risk of multi-system malignancies. Surveillance recommendations are outlined in Table 1 [4]. Colonoscopy is recommended for CRC screening, with some guidelines suggesting a starting age of 15, with screening every 2–3 years [3]. In contrast, newer research suggests a starting age of 35–40, or 10 years prior to the earliest known CRC diagnosis in the family, with suggestions that a normal colonoscopy would not necessitate any different screenings protocols compared to the general population [4, 27, 44]. Colectomy is typically reserved for patients with CRC or those for whom polypectomy is not possible [4].

## Adenomatous polyposis syndromes—autosomal dominant

### Polymerase proofreading-associated polyposis (PPAP)

#### Diagnosis

PPAP is an extremely rare autosomal dominant syndrome caused by germline mutations in the exonuclease domain of DNA polymerase epsilon (*POLE*) and delta (*POLD1*). This affects their proofreading activity, resulting in DNA repair deficiency [45]. There are no current clinical diagnostic criteria for PPAP, with diagnosis only arising after multigene panel testing (typically performed alongside testing for FAP and MAP in suspected patients with ≥ 20 adenomatous polyposis). PPAP can be associated with café-au-lait macules, childhood medulloblastoma, adolescent GIT polyposis, and non-malignant tumours such as pilomatrixomas [45, 46].

#### Genetics/cancer risk

The most common malignancy in PPAP is colorectal cancer (CRC), with a cumulative incidence reportedly 21–90% and 44–90% for *POLE* and *POLD1* mutations, respectively [47]. *POLD1* also increases the risk of endometrial cancer in women, and there is evidence suggesting associations with ovarian, brain, duodenal, and breast cancers [46, 48, 49].

#### Endoscopic/histological features

Patients typically present with an attenuated polyposis phenotype (10–100 polyps), predominantly adenomatous, indistinguishable endoscopically from other polyposis syndromes [47].

#### Management/surveillance

Colonoscopy is recommended every 1–2 years, with gastroduodenoscopy every 1–3 years, starting at ages 14–25 [47, 49]. There is limited evidence supporting routine endometrial screening, although some centres start at age 40. The risk of breast cancer also remains unclear, with referral to a high-risk breast screening clinic for consideration of mammogram at diagnosis being the current advice [47, 49].

### Oligodontia-colorectal cancer syndrome (ODCRCS)

#### Diagnosis

ODCRCS is a rare autosomal dominant condition featuring colorectal adenomatous polyposis, ectodermal dysplasia (sparse hair growth), and oligodontia (congenital absence of six or more teeth)—a distinctive and early feature that may precede GI symptoms [50]. Less than 25 cases have been reported in the literature and there appears to be wide phenotypic variation between heterozygotes [45]. Diagnosis relies upon genetic testing conducted on clinical suspicion, particularly in patients with oligodontia and unexplained polyposis. Prevalence is estimated to be < 1:1,000,000 [51].

#### Genetics/cancer risk

ODCRCS results from a heterozygous germline mutation in the *AXIN2* gene, which is important for intestinal cell maintenance in that it is necessary for feedback inhibition of the Wnt/β-catenin signalling pathway. Dysregulation in this pathway can contribute to colorectal cancer development. The penetrance for CRC is uncertain but likely moderate to high. ODCRCS has also been associated with melanoma, olfactory neuroblastoma, and caecal and prostate adenocarcinoma [51–53].

#### Endoscopic/histological features

61.5% of ODCRCS observed cases have been recorded to have colonic polyps, which range between 10 and 100 in number and are predominantly adenomatous, though sessile serrated polyps have also been observed.

#### Management/surveillance

Surveillance recommendations suggest colonoscopy starting from the age of 25 to 30 years, repeated every 2–3 years if negative. Upper endoscopy is suggested every 3 years, or more frequently dependent on findings. Colectomy may be needed in cases of significant polyp burden [51–53].

## Adenomatous polyposis syndromes—autosomal recessive

### MUTYH-associated polyposis (MAP)

#### Diagnosis

MAP is an autosomal recessive adenomatous polyposis syndrome which shares some clinical similarities with FAP; however, it is typically characterised by the presence of fewer polyps (< 100) and a later age of onset (usually in the 4–5th decade of life) [48, 54, 55]. Polyps are often accompanied by extracolonic features such as duodenal adenomas and sebaceous gland lesions [56]. After FAP, MAP represents the second most common inherited adenomatous polyposis syndrome, with an estimated prevalence of 1:20,000–1:60,000 [45]. Diagnosis is confirmed by the detection of biallelic germline mutations in the *MUTYH* gene in individuals who meet any of the following criteria: ≥ 10 metachronous colorectal adenomas OR a history of adenomas AND extracolonic features (including duodenal/ampullary adenomas, desmoid tumours, thyroid cancer, congenital retinal pigment epithelium hypertrophy, epidermal cysts, and osteomas) [55].

#### Genetics/cancer risk

Biallelic mutations in *MUTYH*, a key gene in the DNA base excision repair (BER) pathway, lead to accumulation of somatic mutations in key driver genes such as *APC*, *KRAS*, and *TP53* [3, 55]. This predisposes to adenoma formation and increases CRC risk by 28-fold [57]. Given its autosomal recessive inheritance, a family history of CRC is often absent [3, 55, 58]. Approximately 1–2% of the general population carry a monoallelic *MUTYH* mutation, whereas biallelic variants are identified in < 1% of CRC cases [59]. The lifetime CRC risk for individuals with MAP is 43–63%, and the risk of duodenal malignancy is 1–6%. Evidence for increased risk of ovarian, bladder, skin, breast, and thyroid malignancy is emerging but remains incompletely defined [19, 27, 50, 55, 56, 60].

#### Endoscopic/histological features

Polyps may occur throughout the colon and are primarily adenomatous, though SSLs and HPs may also be observed [3].

#### Surveillance/management

Colonoscopy should commence at 18–20 years and be repeated every 1–2 years [10, 48, 50, 56, 61]. Upper GI endoscopy is recommended from 20 to 35 years, at 1–3-year intervals. Siblings of affected individuals have a 25% chance of being affected, and first-degree relatives should begin colonoscopy at 45 years, every 5 years [10, 54, 56, 60]. There are no established guidelines for extra-intestinal cancer screening, and screening for monoallelic carriers is not currently recommended due to uncertain CRC risk [10, 54, 56]. Endoscopic polypectomy is recommended for all polyps > 5 mm, with colectomy to be considered in cases of CRC or unmanageable polyp burden [27].

### NTHL1 tumour syndrome (NTS)

#### Diagnosis

*NTHL1* tumour syndrome is an autosomal recessive multi-tumour predisposition syndrome, typically presenting with multiple primary malignancies before age 50 years. It is associated with breast, colorectal, urothelial, endometrial, and haematological cancers, as well as meningiomas, head and neck squamous carcinomas, and basal cell carcinomas. Suspicion should arise in patients with early-onset CRC, or those with ≥ 10 cumulative colorectal adenomas by age 60, or ≥ 20 combined adenomas, hyperplastic, and/or sessile serrated polyps at any age.

#### Genetics/cancer risk

This syndrome is caused by biallelic germline mutations in *NTHL1*, which encodes a DNA glycosylase also integral to the BER pathway. Loss of function results in defective excision of oxidised pyrimidines, leading to somatic C > T transitions and genomic instability. It has a broader multi-tumour spectrum in comparison to MAP. NTS confers a cumulative lifetime extracolonic cancer risk of 35–78%. The risk for CRC is likely high, though not clearly defined [50, 62–64].

#### Endoscopic/histological features

Polyps are predominantly adenomatous, occasionally mixed with serrated lesions. Two to 200 polyps have been observed in the literature.

#### Management/surveillance

Recommended surveillance includes colonoscopy with polypectomy every 2 years from age 18 to 20 years and upper endoscopy every 5 years starting at 25 years. Women should undergo breast imaging from 30 years (1–2 yearly), and endometrial sampling/TVUS from 40 years (2 yearly). Baseline brain MRI should be considered at time of diagnosis given CNS tumour risk. Evidence for other organ-specific screening remains sparse [50, 62–64].

### MBD4-associated neoplasia syndrome (MANS)

#### Diagnosis

*MBD4*-associated neoplasia is a rare autosomal recessive syndrome characterised by adenomatous polyposis, early-onset CRC, and associated with acute myeloid leukaemia/myelodysplastic syndrome, uveal melanoma, and schwannomas. Suspicion should arise and germline *MBD4* testing should be pursued in young CRC patients with microsatellite-stable tumours negative for *APC*, *MUTYH*, and *MMR* variants [47, 50, 65].

#### Genetics/cancer risk

*MBD4* also encodes a DNA glycosylase integral to the BER pathway—one that recognises and removes thymine/uracil mispaired with guanine at methylated CpG sites. Biallelic inactivation produces ultra-mutated, microsatellite-stable tumours with mutational signatures 1 and 30. CRC may present before age 30, alongside myeloid and ophthalmologic neoplasms.

#### Endoscopic/histological features

MANS typically presents with 50–200 polyps, predominantly adenomatous, distributed throughout the colon.

#### Management/surveillance

Colonoscopy every 2 years from age 18 to 20 is recommended. Annual full blood count monitoring is advised for early detection of haematologic malignancy. Ophthalmologic surveillance for uveal melanoma is recommended annually [47, 50, 65].

### MSH3-associated polyposis

#### Diagnosis

*MSH3*-associated polyposis is a rare autosomal recessive syndrome, only very recently described in the literature in 2016. It presents with 10–100 colorectal adenomas, typically arising in the third decade of life.

#### Genetics/cancer risk

*MSH3* encodes a mismatch repair protein that forms the MutSβ complex (with *MSH2*), primarily responsible for repairing insertion/deletion loops in repetitive DNA. Biallelic germline mutations impair this process, leading to elevated microsatellite alterations at selected tetranucleotide repeats (EMAST). This is distinct from classical MSI, so tumours may retain normal MMR immunohistochemistry. Limited data suggest possible associations with breast cancer and duodenal adenomas [66–69].

#### Endoscopic/histological features

*MSH3*-associated polyposis presents with 10–100 polyps, predominantly right-sided and adenomatous.

#### Management/surveillance

Management aligns with attenuated adenomatous polyposis syndromes, including colonoscopy every 1–2 years from 18 to 20 years. There is insufficient evidence to define surveillance guidelines for extra-intestinal malignancies at this stage [66–69].

### MLH3-associated polyposis

#### Diagnosis

*MLH3*-associated polyposis is a rare autosomal recessive adenomatous polyposis syndrome, with first reports in the literature from 2018. It typically presents with 50–200 adenomas and CRC diagnosed in the 4th–5th decade of life. Few studies have reported that it may be associated with infertility and breast cancer [70, 71].

#### Genetics/cancer risk

*MLH3* encodes a MutLγ complex component within the MMR pathway. Biallelic loss of function variants (often somatic) result in microsatellite-stable adenomatous polyposis with chromosomal instability, representing a phenotype distinct from LS. Fewer than 20 affected individuals have been described worldwide [70, 71].

#### Endoscopic/histological features

Fifty to 200 are predominantly adenomatous polyps.

#### Management/surveillance

Given the paucity of data, no specific surveillance guidelines are currently established, though colonoscopic surveillance aligning with the other adenomatous polyposis syndromes is encouraged.

## Mismatch repair deficiency-related syndromes

### Constitutional mismatch repair deficiency (CMMRD)

#### Diagnosis

CMMRD is an extremely rare recessive cancer predisposition syndrome characterised by the absence of functional DNA MMR activity from birth. Consequently, affected patients are highly predisposed to developing multiple malignancies in childhood, including gliomas, colorectal and small bowel cancers, leukaemias, lymphomas, and endometrial carcinomas. Cutaneous and developmental features such as multiple café-au-lait macules, pilomatrixomas, developmental venous anomalies, and immunoglobulin deficiencies are common and may lead to misdiagnosis as neurofibromatosis type 1 [72].

#### Genetics/cancer risk

CMMRD results from biallelic germline mutations in one of the four core *MMR* genes (*MLH1*,* MSH2*,* MSH6*, or* PMS2*), brought together by consanguineous parents. This results in constitutional MSI and markedly increased cancer susceptibility across haematological, neurological, and gastrointestinal systems [73]. The European Consortium Care for CMMRT (2014) developed a diagnostic scoring system incorporating childhood malignancy, cutaneous stigmata, and consanguinity to identify patients warranting genetic testing [74].

#### Endoscopic/histological features

CMMRD may present with adenomatous polyposis resembling attenuated FAP or with multiple early-onset colorectal carcinomas. Histology typically shows MSI-high tumours with loss of MMR protein expression.

#### Management/surveillance

While there is no international consensus on screening, several expert groups have proposed comprehensive surveillance protocols based on the current estimates of malignancy risk and the early age of onset for the multitude of cancers. Annual colonoscopy should begin from age 6 to 8 and annual gastroscopy from age 8 to 10 [72, 75–77]. Additionally, 6-monthly brain MRIs should be considered from age 2 or age of diagnosis, to assess for high-grade gliomas, neuroectodermal tumours, and medulloblastomas [75, 77]. Guidelines have also suggested complete blood counts and abdominal ultrasounds every 6 months from ages 1 to 2 [72]. If multiple colorectal adenomas are found on colonoscopy, preventative colectomy should be considered.

## Mixed polyposis syndromes

### Hereditary mixed polyposis syndrome (HMPS)

#### Diagnosis

HMPS is a rare autosomal dominant syndrome characterized by fewer than 15 mixed-type colorectal polyps (including hamartomas, adenomas, SSLs, juvenile, and mixed polyps) distributed throughout the colon [3, 4]. The average age of polyp detection is 28 years, with no known extracolonic manifestations [78].

#### Genetics/cancer risk

HMPS is most commonly associated with duplication upstream of the *GREM1* gene, leading to *GREM1* overexpression, a BMP pathway antagonist. This results in dysregulated epithelial proliferation and tumorigenesis. This has been observed particularly in individuals of Ashkenazi Jewish ancestry but can occur in other populations. Genetic testing for germline *GREM1* mutations could be considered in families with both adenomas and hamartomas of unknown cause.

#### Management/surveillance

Due to the limited number of reported cases, management strategies are largely extrapolated from FAP guidelines. Colonoscopy surveillance is recommended, though the optimal starting age and interval remain undefined [78, 79].

## Discussion

CRC remains a major contributor to global cancer-related morbidity and mortality, with a significant subset attributable to inherited polyposis syndromes. Though these conditions are individually rare, their cumulative contribution to hereditary CRC is substantial, underscoring the importance of early identification and structured surveillance. Despite significant progress in defining their molecular and clinical features, critical gaps in knowledge persist. Data on long-term outcomes are lacking, particularly in regard to the rare polyposis syndromes.

More case numbers and further studies are required to better understand genotype-phenotype correlations, true malignancy risk, and associations. While many causative mutations have been identified, the nuances of their genotypic and phenotypic heterogeneity are poorly understood. Comprehensive widespread uptake of germline testing when indicated is necessary to further elucidate these nuances. 

Tumours demonstrating high MSI require further testing for *BRAF* mutation to distinguish sporadic cancers from hereditary syndromes. A positive *BRAF* mutation suggests a sporadic origin, whereas *BRAF*-negative tumours may warrant subsequent germline testing for genes associated with LS. In other instances where multigene panel is indicated, multigene panel testing should include *APC* and *MUTYH*, along with other relevant genes implicated in polyposis syndromes such as *POLE*, *POLD1*, *NTHL1*,* AXIN2*,* MBD4*, and *MMR* genes (*MLH1*,* MSH2*,* MSH6*,* PMS2*,* MSH3*, and *MLH3*). Genes associated with hamartomatous syndromes (*STK11*, *BMPR1A*,* SMAD4*,* PTEN*, and *RNF43*) should also be included. Most guidelines recommend multigene panel testing for individuals with ≥ 10–20 polyps identified on cumulative colonoscopy, yet this review indicates that strict adherence to this threshold may cause some of the rarer polyposis syndromes to be missed. A comprehensive clinical assessment, including extra-intestinal features, family history, and phenotype-driven suspicion, is essential. Cascade testing should be offered to relatives of individuals with autosomal dominant syndromes.

Endoscopic surveillance is the cornerstone of managing these syndromes, though there is variability in screening methods and intervals. A personalised approach informed by genotype and phenotype is ideal. Emerging technologies such as chromoendoscopy and artificial intelligence–assisted polyp detection hold promise for improving our polyp detection rates and may help improve the quality of our surveillance regimens.

Many syndromes also involve significant extracolonic manifestations, including malignancies, neurological complications, and vascular anomalies. While some guidelines offer screening protocols for these, further research is needed, especially considering emerging findings about organ-specific cancer risks. Longitudinal studies tracking CRC incidence, extra-intestinal cancers, and quality of life are critical, as is exploring the psychological burden of living with genetic polyposis syndromes and its impact on both the patient and their family members who may also be affected.

The use of chemoprophylaxis, such as NSAIDs or COX inhibitors, has been suggested for some polyposis syndromes, but evidence is still insufficient. Targeted molecular therapies based on specific genetic pathways may represent the next frontier in personalised disease modulation.

Given the growing number of patients being affected by the “rarer” syndromes, a dedicated multi-disciplinary clinic is needed to provide the level of personalised care that is often required for patients presenting with a multitude of colonic and extracolonic manifestations. Patients will require a wide range of investigations and surveillance tools to monitor their ongoing risk of multiple malignancies, and it is vital to ensure that they consistently have access to the best available care based on the most up-to-date guidelines and research.

It is important to acknowledge the limitations of this paper as a narrative review. While attempts were made to follow a systematic approach to assess the articles included for discussion, a formal systematic review was ultimately not feasible due to the breadth of the topic covered which would have been restricted by using a limited search criterion. Due to the lack of high-quality primary studies among the existing literature, additional searches to source information outside of the systematic approach were required for rigorous yet broad review of this topic. However, as a non-systematic review, this article is inherently subject to potential selection bias, as studies were not identified through a fully reproducible search strategy. The lack of standardised inclusion criteria and formal quality appraisal may affect the reliability of the findings. Conclusions drawn should be interpreted with caution, and it is possible that relevant studies were not identified or included.

Guidelines available from larger international jurisdictions (such as the NCCN, Genturis, and American Association for Cancer Research) are often heterogeneous in nature [80–82]; their contents are summarised in Supplementary Table 1. We also provide an overview of the limited advice relating to surgical thresholds provided by these jurisdictions in Supplementary Table 2. Many of these jurisdictions admit that their recommendations are based on guidelines of similar syndromes, with very little evidence or primary studies to support this, further underscoring the need for ongoing investigation to create standardised protocols supported by high-quality data.

Rarer polyposis syndromes, though less common than familial adenomatous polyposis (FAP) and Lynch syndrome (LS), contribute significantly to familial colorectal cancer burden. Continued molecular discovery, collaborative registries, and tailored management frameworks are essential to improving outcomes for this expanding patient group.

## Supplementary Information

Below is the link to the electronic supplementary material.

ESM 1 (18.9 KB DOCX)

## Data Availability

All data generated or analysed during this study are included in this published article.
