# Supplementary material for: The ‘other’ colonic polyposis syndromes—evolving insights and guidance for endoscopists
Source: Int J Colorectal Dis. 2026 Jun 25;41(1):146. doi: 10.1007/s00384-026-05186-8 (PMC13553667; doi:10.1007/s00384-026-05186-8)
Supplement: Supplementary file 1 [file 384_2026_5186_MOESM1_ESM.docx]

**Supplementary Table 1: Comparison of Colonoscopy and Surveillance Guidelines from Large International Organisations**

| **Syndrome** | **NCCN (2025/2026 Version) [80, 81]** | **Genturis^1^ [44, 77]** | | **AACR (2024)^2^ [82]** | **BSG (2019) [10]** | **ESGE (2019) [15]** |
| --- | --- | --- | --- | --- | --- | --- |
| Serrated Polyposis Syndrome | Colonoscopy + polypectomy until all polyps ≥ 5mm removed  1-3 yearly colonoscopy thereafter depending on number and size of polyps  FDR- start at age 40 or same age as youngest diagnosis of serrated polyposis in the family, or 10 years earlier than diagnosis of CRC- whichever is earliest | Genturis- endorses the BSG and ESGE guidelines | | Colonoscopy- start from age 40 or from 10 years earlier than the first diagnosis in the family | 1-2 yearly colonoscopy from diagnosis until age 75  FDR- from age 40 (or 10 years earlier than index case)- 5 yearly colonoscopy until age 75  *Weak-moderate evidence, strong recommendation | Colonoscopy- for polypectomy  Nil starting age  1 year interval- after ≥1 advanced polyp or ≥ 5 nonadvanced  clinically relevant polyps  2-year interval- no advanced polyps, or <5 non-advanced clinically relevant polyps |
| Peutz-Jeghers Syndrome | Baseline colonoscopy at age 8-10 (earlier if symptoms)  If polyps found- repeat every 2-3yrs  If nil polyps- repeat at age 18, then every 2-3yrs thereafter | Genturis- endorses the BSG and ESGE guidelines | | Colonoscopy at age 8 at baseline  If nil polys- repeat around 18yrs  2–3-year frequency | Colonoscopy at age 8 at baseline  If normal, defer until age 18  3 yearly scopes if polyps present  Low evidence, weak recommendation | Colonoscopy- baseline at 8 years (every 1-3 years if polyps found)  Routine from 18 years- every 1-3 years thereafter |
| Juvenile Polyposis | Colonoscopy- start at age 12-15  If polyps found- repeat every 2-3 years depending on findings  If no polyps- resume at 18 years, every 2-3 years thereafter | Genturis- endorses the BSG and ESGE guidelines | | Colonoscopy  Starting age 12-15 years  Annually- can do every 3 years if no polyps  Start earlier if symptomatic | Colonoscopy from age 15, earlier if symptomatic  Interval- every 1-3 years, personalised according to colorectal phenotype  Low evidence, weak recommendation | Colonoscopy  Starting age 12-15  Every 1-3 years |
| Cowden’s Syndrome | Colonoscopy starting age 35  OR 5-10 years before earliest known CRC in family  Frequency- at least every 5 years, more frequently if symptomatic of polyps found | 2024 Guidelines  Guidelines for PHTS  Baseline colonoscopy at 35-40 years to assess polyp load  If baseline scope is normal- do not need screening different to general population | |  |  |  |
| Polymerase Proofreading-Associated Polyposis | Colonoscopy from age 25-30  Every 2-3yrs if negative  Every 1-2 years if polyps found  * Strong evidence |  | |  |  |  |
| Oligodontia-colorectal cancer syndrome | Colonoscopy from age 25-30  Every 2-3yrs if negative  Every 1-2 years if polyps found  Consider surgery if polyp burden unmanageable  * Limited evidence |  | |  |  |  |
| *MUTYH*-Associated Polyposis | Colonoscopy- no later than 25-30, earlier depending on family hx  Every 1-2 years thereafter | Genturis- endorses the BSG and ESGE guidelines, + British Journal of Surgery (2024)  Colonoscopy- from 18 years  Every 1-2 years | | Colonoscopy from 25-30 years | Colonoscopy- from 18-20 years  Annual surveillance  Not recommended for monoallelic  Moderate evidence, strong recommendation | Colonoscopy- from 18 years  Every 1-2 years depending on polyp burden  No surveillance needed for monoallelic patients |
| *NTHL1* Tumour Syndrome | Colonoscopy from age 25-30  Every 2-3yrs if negative  Every 1-2 years if polyps found  * Limited evidence |  | | Colonoscopy- start 25-30 years |  |  |
| *MBD4*-Associated Neoplasia Syndrome | Colonoscopy from age 18-20  Every 2-3yrs if negative  * Limited evidence |  | | Colonoscopy- start 18-20 years |  |  |
| *MSH3*-Associated Polyposis | Colonoscopy from age 25-30  Every 2-3yrs if negative  Every 1-2 years if polyps found  * Limited evidence |  | |  |  |  |
| *MLH3*-Associated Polyposis | Colonoscopy from age 25-30  Every 2-3yrs if negative  Every 1-2 years if polyps found  * Limited evidence |  | |  |  |  |
| Constitutional Mismatch Repair Deficiency |  | 2024 Guidelines  Colonoscopy from age 6  Frequency- annual, every 6 months if polyps detected | |  |  |  |
| Hereditary Mixed Polyposis Syndrome | Colonoscopy from age 25-30  Every 2-3yrs if negative  Every 1-2 years if polyps found  * Limited evidence |  | | Colonoscopy- start 20-25 years |  |  |
| **Abbreviations:**  AACR- American Association for Cancer Research  BSG- British Society of Gastroenterology  ESGE- European Society of Gastrointestinal Endoscopy  NCCN- National Comprehensive Cancer Network | | | **Legend:**  1 Genturis specific guidelines were found for CMMRD & Cowden’s syndrome. Other information listed in the Genturis column was sourced from other guidelines which have been formally endorsed by Genturis.  2 Most AACR data is sourced from NCCN guidelines | | | |

**Supplementary Table 2: Indications for Surgical Consideration from Large International Organisations**

| **Syndrome** | **NCCN (2025/2026 Version) [80, 81]** | | **ACG (2015 Version) [3]** |
| --- | --- | --- | --- |
| Serrated Polyposis Syndrome | Consider surgical referral if colonoscopic treatment/surveillance is inadequate | | Colectomy and ileorectal anastomosis: indicated when unable to control polyp growth, or if cancer present |
| Peutz-Jeghers Syndrome |  | |  |
| Juvenile Polyposis | Colectomy: if polyp burden or polyp related symptoms are not manageable endoscopically or prevent optimal surveillance ^a^ | | Colectomy + IRA or proctocolectomy + IPAA: for polyp-related symptoms, or when the polyps cannot be managed endoscopically |
| Cowden’s Syndrome |  | |  |
| Polymerase Proofreading-Associated Polyposis | Consider surgery if polyp burden becomes unmanageable ^a^ | |  |
| Oligodontia-colorectal cancer syndrome |  | |  |
| *MUTYH*-Associated Polyposis | Total colectomy with IRA if unable to be handled endoscopically- >20 polyps on examination, previous history of polyp ablation, any polyps >1cm, or when advanced histology is encountered  Extent of colectomy dependent on burden and distribution of adenomas  Consider proctocolectomy with IPAA if dense rectal polyposis not manageable with polypectomy | | Colectomy  Absolute indications: documented or suspected cancer or significant symptoms  Relative indications: multiple adenomas >6 mm, significant increase in adenoma number, inability to adequately survey the colon due to multiple diminutive polyps |
| *NTHL1* Tumour Syndrome | Consider surgery if polyp burden becomes unmanageable ^a^ | |  |
| *MBD4*-Associated Neoplasia Syndrome |  | |  |
| *MSH3*-Associated Polyposis | Consider surgery if polyp burden becomes unmanageable ^a^ | |  |
| *MLH3*-Associated Polyposis | Consider surgery if polyp burden becomes unmanageable ^a^ | |  |
| Constitutional Mismatch Repair Deficiency |  | |  |
| Hereditary Mixed Polyposis Syndrome | Consider surgery if polyp burden becomes unmanageable ^a^ | |  |
| **Footnote:** No consensus quantitative polyp threshold has been established for surgical intervention in most non-FAP polyposis syndromes. As demonstrated in this table, contemporary NCCN and ACG guidelines generally do not provide validated polyp-number cutoffs for colectomy/proctectomy in these rare conditions. Instead, recommendations are typically based on qualitative clinical factors, including (i) inability to maintain endoscopic clearance or surveillance, (ii) progressive or dense polyp burden, (iii) refractory bleeding or anaemia, (iv) high-grade dysplasia, (v) colorectal cancer diagnosis, and/or (vi) patient-specific considerations such as polyp distribution, symptom burden, and surveillance feasibility. | | | |
| **Abbreviations**:  ACG- American College of Gastroenterology  IPAA- Ileal pouch-anal anastomosis (aka J pouch surgery)  IRA- Ileorectal anastomosis  NCCN- National Comprehensive Cancer Network | | **Legend:**  a No information/explanation available regarding what defines an unmanageable polyp burden | |
